# Assessing the extent and quality of documentation of fidelity to the implementation strategy: a proposed scoring mechanism

**DOI:** 10.1186/1748-5908-10-S1-A17

**Published:** 2015-08-20

**Authors:** Jennifer N Hill, Erna Snelgrove-Clarke, Susan E Slaughter

**Affiliations:** 1 Spinal Cord Injury Quality Enhancement Research Initiative, Department of Veterans Affairs, Hines, IL, 60141, USA; 2 Center of Innovation for Complex Chronic Health Care, Department of Veterans Affairs; Hines, IL, 60141, USA; 3 School of Nursing & Department Obstetrics/Gynecology, Dalhousie University, Nova Scotia, B3H 4R2, Canada; 4 Faculty of Nursing, University of Alberta, Edmonton, Alberta, T6G 2R3, Canada

## Background

Implementation fidelity is critical to the internal and external validity of a study. Without it, accurate conclusions about an intervention cannot be drawn as unknown factors may have influenced the outcome(s). This scoping review assesses the extent and quality of documentation of fidelity in implementation studies.

## Methods

Articles in the Cochrane Database of Systematic Reviews, Effective Practice and Organization of Care (EPOC) that included at least one of the 47 Cochrane Review implementation strategies were retrieved yielding 1158 articles in 337 journals. We selected publications from the 3 journals with the largest number of articles: British Medical Journal (BMJ), Journal of the American Medical Association (JAMA), and Medical Care. We developed an "Implementation Strategy Fidelity Score" based on definitions adapted from Dusenbury (2003) [1]. Adherence (documenting implementation strategies and extent to which they took place), dose (proportion of people receiving each strategy) and participant responsiveness (extent to which individuals were involved in the development or evaluation of strategies or their receptivity to them) were scored on a 3-point scale: 0=no detail, 1=some detail, and 2=great detail.

## Results

The number of articles included for each journal and mean scores (standard deviations) across categories were: BMJ: n = 28; adherence = 0.86 (SD = 0.87); dose = 0.57 (SD = 0.86); participant responsiveness = 0.79 (SD = 0.90). JAMA: n = 26: adherence = 0.88 (SD = 0.95); dose = 0.76 (SD = 0.81); participant responsiveness = 0.92 (SD = 0.93). Medical Care: n = 19; adherence = 1.11 (SD = 0.97); dose = 0.95 (SD = 1.0); participant responsiveness = 1.21 (SD = 0.89). Although mean scores for the Medical Care journal were higher in each category, documentation of fidelity to the implementation strategy overall can be improved by adopting standard definitions, and better practices for measurement, documentation and reporting.

## Conclusion

This scoring mechanism provides insight into the documentation of fidelity. It also provides a structure for defining and documenting fidelity to the implementation strategy and a benchmark from which to measure the quality of documentation.
